# Development and Assessment of the Validity and Reliability of the Short-Form Life Satisfaction Index (LSI-SF) among the Elderly Population

**DOI:** 10.3390/jpm12050709

**Published:** 2022-04-28

**Authors:** Pei-Shan Li, Chia-Jung Hsieh, Eva Berthy Tallutondok, Ya-Ling Shih, Chieh-Yu Liu

**Affiliations:** 1 Department of Nursing, Taipei Veterans General Hospital, Taipei 112303, Taiwan; phli2@vghtpe.gov.tw; 2 School of Nursing, College of Nursing, National Taipei University of Nursing and Health Sciences, Taipei 112303, Taiwan; eva.tallutondok@uph.edu (E.B.T.); tiffanysyl0902@gmail.com (Y.-L.S.); 3 Faculty of Nursing, Universitas Pelita Harapan, Kota Tangerang 15811, Indonesia; 4 Department of Health Care Management, National Taipei University of Nursing and Health Sciences, Taipei 112303, Taiwan; chiehyu@ntunhs.edu.tw

**Keywords:** life satisfaction, Life Satisfaction Index, validity, reliability

## Abstract

Background: Elderly care should focus on not only prolonging life but also satisfaction with elderly life. Our study investigated the reliability and validity of the Short-Form Life Satisfaction Index (LSI-SF). Method: Data were drawn from the 2015 Taiwan Longitudinal Study on Aging. Internal consistency reliability was used to confirm that the items measured the targeted characteristics. Construct validity was established by confirmatory factor analysis (CFA). Criterion-related validity was examined with the WHO-5 Well-Being Index as an indicator of quality of life. Known-group validity was determined from the difference between frailty stage and quality of life. Results: The high consistency reliability supported the reliability of the LSI-SF. Rigorous CFA validated the construct validity of the LSI-SF. Perfect convergent and discriminant validity supported the validity of the LSI-SF. In addition, there was a significant correlation between the LSI-SF and the WHO-5 Well-Being Index. The LSI-SF appears to be a reliable measure of quality of life in the elderly. We found that frailty status was associated with lower life satisfaction, which supported the known-group validity. Life satisfaction was highest in the non-frailty stage and lowest in the frailty stage. Conclusions: The LSI-SF appears to be a valid and reliable measure of satisfaction with elderly life.

## 1. Introduction

The world’s population is aging, and populations in many countries are also aging [1]. Aging is an inevitable process of life that leads to the progressive failure of organ functions. The physiological phenomena also cause pain and discomfort, which lead to inconvenience in life. While a decline in physiology will occur as a result of aging, aging is an important factor that affects people psychologically [2]. Elderly care should focus more on satisfaction with elderly life than just prolonging their age [3].

Life Satisfaction (LS) represents a subjective evaluation of the current overall status of one’s life [4]. LS can include one’s emotional responses, character, sense of achievement, personal well-being, contentment with one’s job or interpersonal relationships, etc. [5]. Similarly, quality of life (QoL) also presents a subjective experience of the overall status of life [6]. Research shows that both LS and QoL can reflect the physiological, psychological, and social status of the elderly in the community [7]. In older adults, the concept of LS is similar to that of QoL [8]. As the research has already mentioned, LS and QoL are complementary [9].

### 1.1. ‘Life Satisfaction’ Measurement in the Elderly

LS in the elderly includes satisfaction with their living conditions, subjective general well-being, and subjective fulfillment in the dimensions of life [10]. This is different from the previous opinion that only poor health induces low LS. LS has different definitions and broad interpretations based on the respective country’s culture and lifestyle [4]. The instruments for measuring LS focused only on health and mental status in the past [11]. They have since been expanded to include concepts such as values and living environments, as well as being linked to socioeconomic levels and various dimensions [12]. This resulted in no common dimensions within LS measurement. LS is commonly defined as the acceptance of living at certain standards, and QoL can be seen differently [13]. For the older population, LS represents an indicator of QoL [9]. The study pointed out that LS in the elderly is regarded as an important indicator of QoL and the most significant determinant of QoL in the elderly [14]. A systematic review has used the WHO-5 Well-Being Index as a standard to measure QoL as a representation of the elderly [15]. Another study validated that the correlations were significant between QoL (WHO-5 Well-Being Index) and LS scores in the elderly [16].

LS measures must be developed for specific conditions, populations, or functional states. As a result, different measures have different dimensions and items. In 1961, Neugarten et al. aimed to measure the mental health of the elderly and evaluate the effects of successful aging. They derived and validated the Life Satisfaction Rating (LSR). To do so, they developed a 20-item version of the Life Satisfaction Index-A (LSIA) ‘for middle-aged and older adults in the United States’ [5] to assess the well-being of older adults [17]. The five dimensions of the LSIA are ‘Zest vs. Apathy’, ‘Resolution and Fortitude’, ‘Congruence Between Desired and Achieved Goals’, ‘Self-Concept’ and ‘Mood Tone’. The items of the LSIA were scored on a three-point scale of ‘agree’, ‘disagree’, or ‘don’t know’. Scholars have confirmed that in the LSIA, LS is multifaceted rather than a single concept [18]. In 1969, Adams conducted a factor analysis of 1716 elderly in the community to confirm the dimensions of the LSI-A. The results showed that the Life Satisfaction Index-11 (LSI-11) questions had a total of 11 items but only four clear dimensions. The LSI-11 dimensions of ‘Emotional Orientation’, ‘Zest vs. Apathy’ and ‘Congruence Between Desired and Achieved Goals’ were correlated, but the fourth dimension combined ‘Congruence Between Desired and Achieved Goals’ and ‘Resolution and Fortitude’, which seemed unclear [19]. This study also found that older adults developed fatigue when they answered too many questions, so Adams developed a 13-item version of the Life Satisfaction Index-Z (LSIZ). It also reduced the responses to ‘agree’ or ‘disagree’ [19].

In 1980, Lohmann selected 25 items from seven LS instruments to define LS and performed factor analysis on different living conditions (institutional/community), disability status, elderly, etc. The results indicated that the LS had two dimensions in the elderly, and the LSI-A items had significant impacts on both dimensions [20]. These findings suggest that LS in older adults is not affected by a single factor. In 1981, the Health Promotion Bureau under the Ministry of Health and Welfare Department of Taiwan selected 10 items from the LSIA to develop the Taiwan Longitudinal Study on Aging-Life Satisfaction Index (TLSA-LSI). It was used to measure the LS of middle-aged and elderly adults, and it also indicated the QoL of the middle-aged and elderly populations in Taiwan [21]. The validity and reliability of the TLSA-LSI have yet to be assessed. In 1984, Liang et al. even performed LSI-11 validation by structural equation modeling on a sample of 2797 older adults and randomly divided them into four subgroups to develop a model with an acceptable fit. The results showed that the overall model fit and the factor loadings of each item were greater than 0.45 and statistically significant, but the average variance extracted (AVE) and composite reliability (CR) were not examined or reported [22]. In addition to this, LS has different dimensions of culture, race, age, and social status [17]. It also affects the well-being of different genders in some countries [23]. It is recommended that the reliability and validity of the LSIA be evaluated in different populations and cultures [17]. 

### 1.2. Research Questions and Purposes

In addition to frailty due to aging, there are increased risks of disease, cognitive decline, and psychological symptoms of depression or anxiety [24]. It is necessary to consider different ethnic or cultural differences in designing LS measures appropriate for older adults [18]. The LSIA and LSIZ have reliability, but not validity. If the instrument can effectively measure LS in older adults, it needs to have both reliability and validity. With this in mind, our study aimed to confirm the reliability of the Life Satisfaction Index in older adults, analyze the internal consistency reliability, test the construct validity by classifying the theoretical dimensions by item type, use the WHO-5 Well-Being Index to test the criterion-related validity, and test the known-group validity according to frailty level. The purpose of our study was to investigate the reliability and validity of the Short-Form Life Satisfaction Index (LSI-SF), applying the characteristics of the elderly and Chinese culture, to show the overall situation of the elderly in Taiwan.

## 2. Methods

### 2.1. Study Design and Data Collection

Our study data were drawn from the Taiwan Longitudinal Study on Aging (TLSA), which was conducted by the Bureau of Health Promotion under Taiwan’s Department of Health. The database was established in 1989 in cooperation with the Taiwan Health Promotion Bureau, the University of Michigan’s Population Research Center, and the University of Michigan’s Institute of Gerontology to provide information on aging in Taiwan. It conducts cohort surveys, creates data, and follows the physiological, psychological, and social status of the middle-aged and elderly populations in Taiwan. The original cohort sampling was based on non-institutionalized participants and a three-stage probability sampling based on the national household registry in Taiwan. The structured questionnaire, which was validated by experts, included characteristics, health status, social support, employment status, leisure and social participation, aging mentality, economic status, etc. Data were collected through individual interviews with participants who completed the training. Data entry, checks and filing were all performed with strict process control to ensure quality [25]. Since TLSA is a nationwide prospective cohort study, the collected data should be able to fully reflect the health and living conditions of middle-aged and older people in Taiwan over the years. The relevant research can be used as a basis for discussion of important issues such as Taiwan’s aging population, elderly health care, and welfare policies. We were to investigate the reliability and validity of the LSI-SF based on the TLSA survey (the 2015 survey). Inclusion criteria were older adults (60 years old and above). Exclusion criteria were (i) severe dementia (i.e., the Short Portable Mental State Questionnaire (SPMSQ) score standard in Taiwan was less than 2 [26].); (ii) in the absence of TLSA-LSI reverse encoding item conversion, the answers for all items remaining the same as before; (iii) missing data on the TLSA-LSI and frailty status. Our study was approved by Fu Jen Catholic University (FJU-IRB No: C110040).

### 2.2. Measures

#### 2.2.1. Background Characteristics of the Participants

We collected demographic data including age, gender, spousal status, and education level.

#### 2.2.2. Short-Form Life Satisfaction Index (LSI-SF)

The LSI-SF was to measure LS in the elderly in Taiwan. The items and dimensions of the TLSA-LSI are based on the theory of LSIA. The three dimensions are ‘Zest vs. Apathy’, ‘Resolution and Fortitude’, and ‘Congruence Between Desired and Achieved Goals.’ It has a total of six items, of which item 2 needs reverse conversion for negative questions (Table 1). This instrument is designed with dichotomous scoring for each item (0: no, 1: yes), and a higher score indicates a higher level of LS. The Cronbach’s alpha ranges from 0.85 to 0.92 [27].

#### 2.2.3. WHO-5 Well-Being Index

The WHO-5 Well-Being Index was developed by the World Health Organization in 1998 [28]. It consists of 5 items rated on a 6-point Likert-type scale. The score range for each item is 0 to 5, with low to high scores representing ‘constantly present’, ‘present at most of the time’, ‘present more than half of the time’, ‘present less than half of the time’, ‘occasionally’, and ‘not present’, respectively. A higher score indicates a higher level of well-being. The Cronbach’s alpha of the Taiwanese version is 0.94. It is an appropriate instrument for measuring QoL in older adults [29]. Therefore, we used the WHO-5 Well-Being Index as an indicator of QoL.

#### 2.2.4. Definition of Frailty

In the first wave of TLSA conducted in 1989, no widely accepted operational definition of frailty was available. Similar modifications have been imitated by the Survey of Health, Ageing, and Retirement in Europe (SHARE) in previous research [30,31]. Similarly, the TLSA researchers have used the same definition of frailty to fit the data, which were selected retrospectively starting with the TLSA’s fifth survey in 2003 [32,33]. Frailty was defined according to the Fried criteria, which list the five characteristics as shrinking, weakness, exhaustion, slowness, and low physical activity. If 3 or more of the five traits are indicated, then frailty is confirmed, and 1–2 traits indicate the pre-frailty stage [34]. Because we retrieved data from TLSA questionnaires, we used a modified definition for these five traits. It has been widely used and validated in the literature [35,36]. We used ‘loss of appetite’ to refer to nutritional status and to indicate ‘shrinking’. Participants who reported frequent loss of appetite in the previous week were classified as shrinking [30,31,32,33,35,36]. Participants who found it difficult to carry 12 kg of groceries were classified as having weakness [30,31,32,33,35,36]. We used the Center for Epidemiologic Studies Depression Scale (CES-D) to define ‘exhaustion’. Participants who answered ‘I could not get going’ or ‘I felt everything I did was an effort’ frequently or most of the previous week were characterized as having exhaustion [30,31,32,33,35,36]. We used walking or moving in and around the house to show gait speed and indicate ‘slowness’. Participants who were unable to walk a distance of 200 to 300 m or found it difficult were classified as having slowness [30,31,32,33,35,36]. We used the frequency of leisure time/physical activity per week to show the level of physical activity to indicate ‘low physical activity’. Participants who did not partake in walking, hiking, jogging, gardening, or other outdoor activities at least once or twice a week were classified as having low physical activity [30,31,32,33,35,36].

### 2.3. Statistical Analysis

Background characteristics and LSI-SF data are presented as numbers and percentages or means and standard deviations for continuous variables. First, the relationship of LSI-SF scores to demographic data such as age, gender, spousal status, education level, and frailty stage were analyzed. Internal consistency reliability was evaluated with Cronbach’s alpha by calculating the Kuder–Richardson 20 value (KR-20) to confirm that the items measured the targeted characteristics. Construct validity was established with a Confirmatory Factor Analysis (CFA) model to test the theory. The model fit evaluation included three aspects: basic fit criteria, overall model fit, and fit of internal structure of model. Model fit was measured by absolute fit measures (χ^2^, RMSEA, GFI, AGFI), relative fit measures (NFI, CFI), and parsimonious fit measures (PNFI, CN). Criterion-related validity was evaluated by the correlation between the LSI-SF and the WHO-5 Well-Being Index to indicate QoL. Known-group validity was assessed by analysis of variance (ANOVA) to explore the difference between frailty stages and QoL. All data were analyzed in SPSS-22 and LISREL-8.80.

## 3. Results

### 3.1. Social–Demographic Status and TLSA-LSI Scores of the Participants

A total of 2321 participants were included in the analysis. The largest group was 65–69 years old (25.50%). Roughly half (50.50%) were female, 64.70% had completed primary education, and 71.30% lived with a spouse. Close to half (46.30%) of the participants were in the pre-frailty stage. Age, gender, education level, spousal status, and frailty status were significantly different on the LSI-SF (Table 2). 

The means of the LSI-SF and WHO-5 Well-Being Index were 4.62 ± 1.77 and 3.86 ± 1.67, respectively. The mean range of each item in the LSI-SF was 0.66 to 0.85. Item 1 had the lowest mean and standard deviation (0.66 ± 0.47), items 2 and 4 had the highest means and standard deviations (0.85 ± 0.36), and almost half of the participants achieved a full score on the LSI-SF (46.8%).

### 3.2. Reliability

We found that the internal consistency reliability of the LSI-SF was very good (Cronbach’s α = 0.81).

### 3.3. Validity

#### 3.3.1. Construct Validity

Since these six items were theoretically loaded with three factors, CFA was performed on the data of the older adults (*n* = 2321), and the same three-factor structure was identified. Maximum likelihood CFA was used to test the goodness-of-fit index (GFI) of the three-factor model. The results showed that the chi-square value of the three-factor structure was significant (χ^2^ = 98.58). From the other GFI indicators, the CFA showed that this model conforms to the criteria (Table 3).

The LSI-SF met the maximum likelihood criteria under the GFI, and the result of the confirmatory factor analysis (CFA) was satisfactory. The internal fit of the LSI-SF was between 0.26 and 0.64 (Table 4). Therefore, the convergent validity and discriminant validity of the LSI-SF were analyzed.

**Figure 1:**
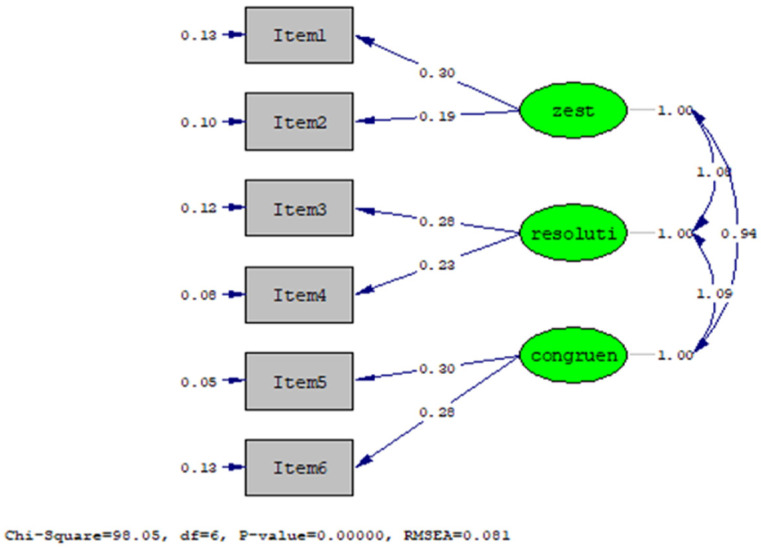
Results from the one-order factor analysis of the LSI-SF.

1. Convergent Validity: Factor Loadings for all items ranged from 0.51 to 0.80 and were significant (α = 0.05) (Table 4, Figure 1). This shows that the items in the LSI-SF can reflect dimensions. In the AVE section, ‘Resolution and Fortitude’ and ‘Congruence Between Desired and Achieved Goals’ were above the norm of 0.50, but ‘Zest vs. Apathy’ was less than the norm of 0.50 (Table 4). 

2. Discriminant Validity: The three dimensions of the LSI-SF had significance (*p* < 0.05), and the confidence intervals of the three dimensions did not contain 1.00 (Table 5).

#### 3.3.2. Criterion-Related Validity

The LSI-SF was significantly associated with the WHO-5 Well-Being Index (r = 0.64; *p* < 0.01).

#### 3.3.3. Known-Group Validity

Frailty status and LSI-SF were analyzed by analysis of variance (ANOVA).

**Figure 2:**
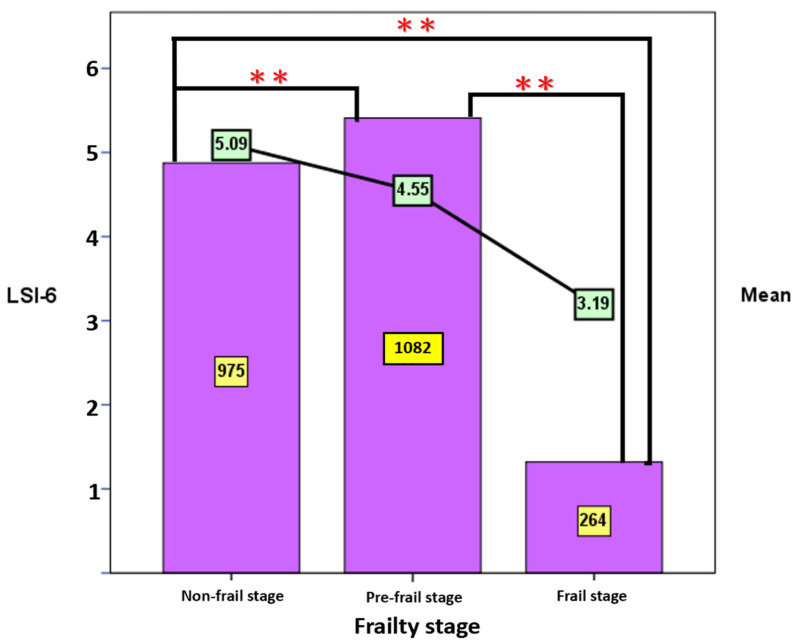
LSI-SF vs. Frailty status. Legend: Non-frailty (*n* = 975), Pre-frailty (*n* = 1082), Frailty (*n* = 264); LSI-SF decreased with increasing frailty status. ** *p* < 0.01.

We found a significant difference between frailty statuses and LS among the elderly (F = 134.89, *p* < 0.01). LS in the non-frailty stage was the highest, and LS in the frailty stage was the lowest (Figure 2). Old adults with the lowest LS may be frail.

## 4. Discussion 

### 4.1. Participant Characteristics and LSI-SF 

In the TLSA 2015 survey, we found that age was significantly associated with LS. Older age was associated with lower LS, which was consistent with the research results from most countries in recent years [37,38,39,40,41,42]. The literature points out that Asian seniors will affect LS under the mental and physical conditions of aging [43]. Older adults experience physical and psychological aging, leading to a decline in overall health, which directly affects their independence and vitality in life [44]. Thus, older adults themselves can gradually perceive a decline in their overall health with age, which could explain the causality of the decline in LS among our population.

We also found that the LS of older women was significantly lower than that of men in the 2015 survey, which is consistent with the findings of Elmstahl et al. [38,39]. We speculated that the elderly were influenced by the patriarchal belief that men are superior to women at a young age, which in turn affected women’s rights in all aspects [23]. In addition, the post-war baby boom pushed women to increase their labor, and women shouldered the double burden of work and family [45]. Under these long-term influences, LS in the elderly was affected by gender differences. Higher education was also associated with increased LS. The education level of the elderly is positively correlated to LS [7,42], and in older adults, higher education is an important indicator of LS [37,39]. Our study showed a significant relationship between spousal status and LS, and previous research also pointed to a negative correlation between LS and lack of a spouse [46]. Greater frailty in an older person was correlated with lower LS, which was consistent with a previous finding that frailty state was negatively correlated with LS [47].

### 4.2. Reliability of the LSI-SF

The internal consistency reliability of the LSI-SF (Cronbach’s alpha of 0.81) was higher than that of the TLSA-LSI (Cronbach’s alpha of 0.73). Therefore, our study found that the LSI-SF has good internal consistency reliability. Enkvist et al. focused on the elderly and found that the Cronbach’s α of the LSI-A was 0.78 [48]. Wylie et al. focused on community-based elderly with the KR-20 and found that the Cronbach’s α of the LSI-Z was 0.79, indicating internal consistency reliability [49], and these conclusions were similar to those of our study. However, Abraham et al. tested the LSI-Z with the KR-20 in frail, multiply disabled, and depressed elderly residents in long-term care institutions 18 times, and the highest Cronbach’s alpha coefficient was only 0.60. This suggested that the reliability of the LSI may be inconsistent in depressed or frail older adults [50].

### 4.3. Validity of the Short-Form Life Satisfaction Index 

#### 4.3.1. Construct Validity

Among the LSI-A-developed milestones, there was no consensus on what constitutes LS, possibly due to differences in sample size and subject homogeneity [5,19,20,22]. In our study, the LSI-SF items were based on the LSI-A and administered to 2348 elderly subjects, according to the three dimensions of ‘Zest vs. Apathy’, ‘Resolution and Fortitude’ and ‘Congruence Between Desired and Achieved Goals’. While the chi-square value in the GFI may not be perfect, the chi-square test is easily limited by the sample size and degrees of freedom. When the sample size is large, the model has the disadvantage of being easily rejected. Therefore, our results showed that the LSI-SF had sufficient construct validity.

Review of previous research findings LS in older adults is influenced by a variety of factors [20], the convergent validity of which is unknown [22]. Construct validity for integrity has also been rarely explored in previous studies [51]. Although in our analysis, not all dimensions met the criteria. According to Fornell et al., if the AVE is less than 0.5 but the composite reliability is higher than 0.60, then the convergent validity of the construct is still sufficient [52]. Our analysis confirmed the convergent validity. For discriminant validity, it showed the differences between the dimensions and items, and the correlation between them was discriminated against. Our analysis indicated that the discriminant validity was acceptable. Therefore, our findings suggest that the LSI-SF has good convergent validity and discriminant validity. It can be seen that LS in the elderly was affected by multiple important factors, such as enthusiasm for life, life goals, and independent living.

#### 4.3.2. Criterion-Related Validity

According to our findings for criterion-related validity, the LSI-SF and WHO-5 Well-Being Index showed acceptable concurrent validity. There was a significant positive correlation between LS and well-being, and LS and well-being in the elderly had a moderate level of correlation in older adults. Well-being is a measure of subjective QoL in the elderly that is also correlated with LS [16,51]. Their QoL will also increase their satisfaction with life [53]. The primary goal should be to understand the key factors affecting LS in older adults and to actively assist the elderly to meet specific life-related needs.

#### 4.3.3. Known-Group Validity

Most studies have pointed to a significant connection of LS with frailty in the elderly [54,55]. Our finding was consistent with previous research showing that higher frailty status is associated with lower LS. Well-being and QoL tend to be lower in the frail elderly. Therefore, according to the needs of the elderly, care planning should be arranged for daily activities to improve mental health and to slow down the progression of frailty.

### 4.4. Limitations

A major limitation of our study was the lack of further differentiation of LS across different states of the elderly. We have not discussed the LS of older adults living in residential institutions. Perhaps ethnicity, time spans, gender, and age groups all contribute to contradictions and inconsistencies in LS in the elderly. The diversity of the results obtained by subgroup analysis can strengthen the clinical utility of the LSI-SF in future research.

## 5. Conclusions

Our results show that the LSI-SF has good psychometric properties. The high consistency reliability supports the reliability of the LSI-SF. Rigorous CFA validated the construct validity of the LSI-SF. The perfect convergent and discriminant validity supported the validity of the LSI-SF. In addition, there was a significant correlation between the LSI-SF and the WHO-5 Well-Being Index, indicating that the LSI-SF can be used to assess QoL in the elderly. The LSI-SF is also an effective indicator of frailty. These results could serve as a reference or be used for comparison in the future to understand the relationship between frailty status and LS. However, comparatively little research has been done on how the disability status and activity level of the elderly affect frailty and LS in the elderly. Further research is needed to identify the main mediating factors in maintaining LS in older adults. Appropriate interventions should then be designed according to the characteristics of older adults and the progression of frailty in the elderly to achieve the goal of successful aging.

## Figures and Tables

**Table 1:** Items and dimensions of the LSI-SF.

Dimension	Item
Zest vs. Apathy	1. These are the best years of my life.
	2. Most of the things I do are boring or monotonous.
Resolution and Fortitude	3. As I grow older, things seem better than I thought they would be.
	4. The things I do are as interesting to me as they ever were.
Congruence Between Desired and Achieved Goals	5. As I look back on my life, I am fairly well satisfied.
	6. I’ve gotten pretty much what I expected out of life.

**Table 2:** Background characteristics and LSI-SF scores of the participants (N = 2321).

	Total		LSI-SF		
	N	%	Mean	SD	F/t
Age					2.93 **
60–64	528	22.70	4.77	1.65	
65–69	592	25.50	4.76	1.67	
70–74	393	16.90	4.63	1.71	
75–79	326	14.00	4.36	2.06	
80–84	217	9.30	4.46	1.85	
≥85	265	11.40	4.46	1.82	
Gender					1.29 **
male	1154	49.70	4.67	1.72	
female	1167	50.30	4.57	1.82	
Education					11.11 **
primary school	1493	64.30	4.48	1.86	
middle school	264	11.40	4.64	1.73	
high school	420	18.10	4.97	1.47	
university	144	6.20	5.02	1.50	
Spousal status					8.28 **
with a spouse	1660	71.50	4.81	1.64	
without a spouse	661	28.50	4.15	1.98	
Frailty status					134.89 **
non-frailty stage	975	42.00	5.09	1.39	
pre-frailty stage	1082	46.60	4.55	1.77	
frailty stage	264	11.40	3.19	2.18	

** *p* < 0.01.

**Table 3:** Fit indices for the performance model of the LSI-SF.

Maximum Likelihood with Correlated Factors		Outcome	Criterion
Absolute Fit Measures	χ^2^	98.58	smaller is better
	RMSEA	0.08	<0.10
	GFI	0.99	>0.90
	AGFI	0.95	>0.90
Incremental Fit Measures	NFI	0.98	>0.90
	CFI	0.98	>0.90
Parsimonious Fit Measures	CN	396.67	≥200

Notes: RMSEA = root mean square error of approximation. GFI = goodness of fit index. AGFI = adjusted goodness-of-fit index. NFI = normed-fit index. CFI = comparative fit index. CN = critical N.

**Table 4:** Reliability and convergent validity of the LSI-SF.

One-Order CFA Factor	Unstandardized Estimate	S.E.	t/p	Standardized Estimate	Factor Loadings	R^2^	AVE	CR
1. Zest							0.43	0.601
Item 1	0.30	0.01	26.16 **	0.63	0.71	0.40		
Item 2	0.19	0.01	22.16 **	0.51	0.60	0.26		
2. Resolution							0.54	0.701
Item 3	0.28	0.01	29.24 **	0.62	0.72	0.39		
Item 4	0.23	0.01	29.77 **	0.63	0.75	0.40		
3. Congruence							0.58	0.733
Item 5	0.30	0.01	39.52 **	0.80	0.82	0.64		
Item 6	0.28	0.01	29.92 **	0.61	0.70	0.38		
criteria					>0.60	>0.20	>0.50	>0.50

Notes: Zest = Zest vs. Apathy. Resolution = Resolution and Fortitude. Congruence = Congruence Between Desired and Achieved Goals. ** *p* < 0.01.

**Table 5:** Correlation coefficients between the dimensions of the LSI-SF.

Dimension	Zest vs. Apathy	Resolution and Fortitude
Resolution and Fortitude	1.08 (33.25 *) [1.02, 1.78]	
Congruence Between Desired and Achieved Goals	0.94 (33.77 *) [0.88, 0.99]	1.09 (48.95 *) [1.05, 1.13]

* *p* < 0.05; ( ) Chi-square difference; [ ] confidence interval.

## Data Availability

The data that support the findings of this study are available from the Health Data Science Center in Taiwan, but access to these data is restricted because they were used under license for the current study and are thus not publicly available. Data is available at https://www.hpa.gov.tw/EngPages/Index.aspx with the permission of the Taiwan Health Promotion Administration on 7 January 2022.

## Abbreviations

LSI-SF	Short-Form Life Satisfaction Index
CFA	confirmatory factor analysis
LS	Life Satisfaction
QoL	quality of life
LSIA	Life Satisfaction Index-A
LSI-11	Life Satisfaction Index-11 questions
LSIZ	Life Satisfaction Index-Z
TLSA-LSI	Taiwan Longitudinal Study on Aging-Life Satisfaction Index
TLSA	Taiwan Longitudinal Study on Aging
KR-20	Kuder–Richardson 20 value
GFI	goodness of fit index
AVE	average variance extracted
CR	Composite Reliability
